# Plants attacked above-ground by leaf-mining flies change below-ground microbiota to enhance plant defense

**DOI:** 10.1093/hr/uhae121

**Published:** 2024-04-26

**Authors:** Yang Gao, Qiong Yang, Qiulin Chen, Yunchuan He, Wei He, Jiamei Geng, Yunzeng Zhang, Ying Zhou, Zeng-Rong Zhu

**Affiliations:** Hainan Institute, Zhejiang University, Sanya, 572025, China; State Key Laboratory of Rice Biology, Institute of Insect Sciences, Zhejiang University, Hangzhou, 310058, China; School of BioSciences, Bio21 Institute, The University of Melbourne, Parkville, VIC 3010, Australia; Hainan Institute, Zhejiang University, Sanya, 572025, China; State Key Laboratory of Rice Biology, Institute of Insect Sciences, Zhejiang University, Hangzhou, 310058, China; Hainan Institute, Zhejiang University, Sanya, 572025, China; State Key Laboratory of Rice Biology, Institute of Insect Sciences, Zhejiang University, Hangzhou, 310058, China; Hainan Institute, Zhejiang University, Sanya, 572025, China; State Key Laboratory of Rice Biology, Institute of Insect Sciences, Zhejiang University, Hangzhou, 310058, China; Hainan Institute, Zhejiang University, Sanya, 572025, China; State Key Laboratory of Rice Biology, Institute of Insect Sciences, Zhejiang University, Hangzhou, 310058, China; Joint International Research Laboratory of Agriculture and Agri-product Safety of the Ministry of Education, Yangzhou University, Yangzhou, 225009, China; Hainan Institute, Zhejiang University, Sanya, 572025, China; Hainan Institute, Zhejiang University, Sanya, 572025, China; State Key Laboratory of Rice Biology, Institute of Insect Sciences, Zhejiang University, Hangzhou, 310058, China

## Abstract

Root-associated microbiomes play a crucial role in plant responses to biotic and abiotic stresses. Plants can enrich beneficial microbes to increase their stress-relieving ability. Above-ground insect herbivory is among the most detrimental stresses for plants, especially to crop production. However, few studies have explored how root-associated microbiomes respond to herbivores and influence plant-defense functions under herbivory stress. We investigate the changes and functional role of root-associated microbial communities under herbivory stress using leafminer (*Liriomyza trifolii*) and cowpea (*Vigna unguiculata*) as a focal system. We did this by using a combination of 16S ribosomal RNA gene profiling and metagenomic sequencing to test for differences in co-occurrence networks and functions between cowpea plants infested and noninfested with leafminers. The results demonstrated that leafminer infestation caused a shift in the rhizosphere microbiome, which was characterized by a significant variation in microbiome community structure and composition, the selection of hub microbes involved in nitrogen (N) metabolism, and functional enrichment related to N metabolism. Notably, nitrogen-fixing bacteria *Bradyrhizobium* species were actively enriched and selected to be hubs in the rhizosphere. Inoculation with *Bradyrhizobium* enhanced cowpea performance under leafminer stress and increased protease inhibitor levels to decrease leafminer fitness. Overall, our study characterized the changes of root-associated microbiota between leafminer-infested and noninfested cowpea plants and revealed the mechanisms underlying the rhizosphere microbiome shift that enhance plant performance and defense against herbivory. Our findings provide further support for the notion that plants enrich rhizosphere microbes to counteract aboveground insect herbivores.

## Introduction

Plants and their microbiota are intricately linked, constituting a holobiont that affects plant fitness [1, 2]. The diversity of plant-associated microbiome in bulk soil, rhizosphere, endosphere, and phyllosphere is immense, significantly expanding the functional repertoire of plants beyond imagination [3–5]. Studies have reported that the plant-associated microbiome can promote plant growth, facilitate nutrient acquisition, promote abiotic stress tolerance, and enhance pathogen resistance [6–9]. Emerging evidence indicates that plants utilize the ‘cry for help’ strategy in the rhizosphere, actively recruiting beneficial microbes to combat bacterial and fungal pathogen [10–13]. Gaining insights into the structure and function of plant-associated microbiome communities under different environmental conditions may provide avenues for establishing novel resource-efficient and stress-resistant agroecosystems [14, 15].

Insect herbivory is among the most detrimental biotic stresses affecting plant health [16]. Previous studies have illustrated how a few key underground microbes can induce a cascade of plant physiological changes that can affect the performance of herbivorous insects (for example, the roles of root endophytic fungi [17], mycorrhizal fungi [18, 19], plant growth-promoting fungi [20] and rhizobacteria [21], and rhizobia [22]). However, while key microbes might influence plant-insect interactions, it is unclear whether plants can generally use the ‘cry for help’ strategy to combat herbivorous insects as they do for pathogens. Some recent studies suggest that insect herbivory can induce changes in plant-associated microbiota, and in some cases, have demonstrated that these changes can feedback to influence the outcome of plant-insect interactions [23, 24]. For example, whitefly (*Bemisia tabaci*) infestation reshaped the rhizosphere microbiota structure of pepper and led to the recruitment of fluorescent *Pseudomonas* [25]. Similarly, aphid (*Macrosiphum euphorbiae*) herbivory altered the rhizosphere microbiome of tomato and the soil legacy affected aphid performance in the next plant generation [26]. Shoot insect herbivores (*Brevicoryne brassicae* and *Plutella xylostella*) and root insect herbivores (*Delia radicum*) could lead to the distinct rhizosphere microbiome of cabbage, with consequences for the plant resistance by plant–soil feedback [27]. In addition, the leafminer *Scaptomyza nigrita*, via induction of plant defences by jasmonic acid (JA), reshaped the phyllosphere endophytic microbiome of native bittercress [28]. These studies suggested that insect herbivory can primarily drive the community dynamics of plant-associated microbiome, but the effects of insect herbivory on microbiome functions and microbial networks have not been studied and whether plants utilize the ‘cry for help’ strategy in response to insect herbivory is still poorly understood. Demonstrating such feedback validates the functional significance of plant microbiome community changes in response to insect herbivory, and the importance of ‘cry for help’ strategies in the outcomes of plant–insect interactions.

In this present study, we study the potential use of an insect herbivory ‘cry for help’ in cowpea (*Vigna unguiculata*) infested by American serpentine leafminer, *Liriomyza trifolii* (Agromyzidae). Cowpea is a high-quality legume protein source consumed worldwide due to its high protein, complementary amino acid profile, and relatively low-fat content compared to cereal grains [29], but infestation by *L. trifolii* can severely limit yield and production [30]. *L. trifolii* female adults puncture the leaf surface and oviposit beneath the epidermis, larvae then tunnel and feed within the leaf, which reduces the plant’s photosynthesis capacity and ultimately its productivity [31]. The application of chemical insecticides is the most widely used method for leafminer control, but improper use of insecticides can lead to the development of resistance in leafminer, contaminated food crops and an increased risk to public health [32]. Therefore, there is an urgent and present need to identify alternatives to chemical insecticides. It has been reported that the underground microbes can induce plant resistance against aboveground insects, which is composed of direct defense such as the production of toxins [21, 33–35] and indirect defense such as emitting plant volatiles that attract natural herbivore enemies [36] or deter insect herbivores [37]. This indicates the root-associated microbes can serve as a potential line of defense to combat aboveground insects, and it should be included in agricultural management strategies [23]. Understanding how the root-associated microbiome responds to leafminer infestation can provide valuable insights for the development of more effective strategies for leafminer control.

Here, we study the potential ‘cry for help’ in the cowpea–leafminer system using 16S amplicon and metagenomic sequencing of root-associated microbiomes of infested and noninfested plants in combination with experimental and molecular assays. We used this data to achieve three study objectives: (1) to characterize changes in the composition and function of root-associated microbiome between the infested and noninfested plants; (2) to identify key microbial members associated with leafminer infestation; and (3) to identify the functional pathways that key microbial members may provide resistance to leafminer infestation.

## Results

### Leafminers affect rhizosphere microbiota assembly

A total of 4 113 369 high-quality sequences were procured from 80 samples, with an average of 51 416 reads per sample (range: 31051–111 533 reads per sample). And these sequences were denoised using DADA2 [38], eliminating chimeric and organelle sequences, resulting in the generation of 7696 ASVs.

**Figure 1:**
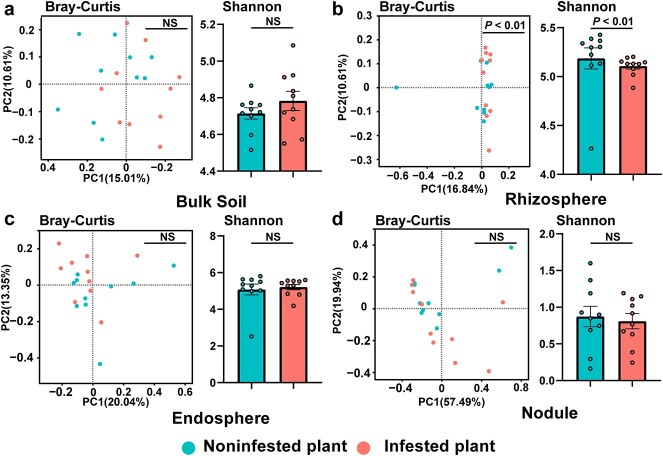
β diversity and α diversity of root-associated microbiota in infested plants and noninfested plants. The different patterns of root-associated microbiota in (**a**) bulk soil, (**b**) rhizosphere, (**c**) endosphere, and (**d**) nodule. β diversity is characterized by unconstrained PCoA with Bray–Curtis dissimilarity matrices and analysed in PERMANOVA. α diversity is characterized by Shannon indices and analysed in Kruskal–Wallis. The data bars represent the means, while the error bars represent the standard error.

**Figure 2:**
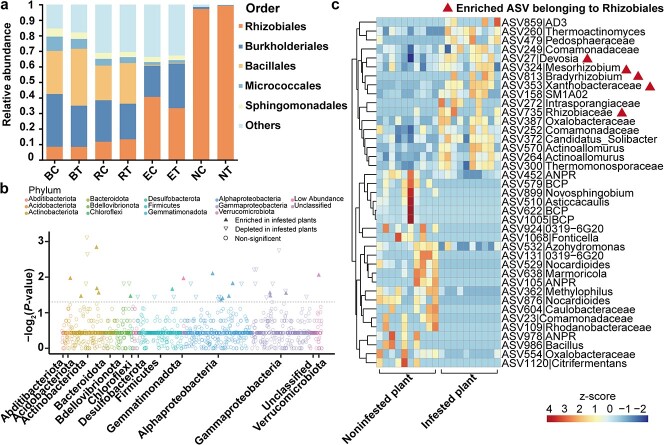
Different taxonomic characteristics of root-associated microbiota between infested and noninfested plants. **a** Order-level community composition in the different samples. B: bulk soil; R: rhizosphere; E: endosphere; N: nodule; C: noninfested plants; T: infested plants. **b** Manhattan plot showing differential ASVs encompassed several phyla in rhizosphere compartment. Differential ASVs are represented by triangles and non-significant ASVs are represented by circles (*P* < 0.05, Wilcoxon rank-sum test). **c** Heatmap showing the relative abundance of differential ASVs in rhizosphere compartments. Color key to heatmap is shown as standardized z-score of ASVs’ relative abundance. *Allorhizobium-Neorhizobium-Pararhizobium-Rhizobium* and *Burkholderia-Caballeronia-Paraburkholderia* were abbreviated to ANPR and BCP, respectively, for visualization.

Our analyses revealed distinct patterns of community structure and microbial diversity among the bulk soil, rhizosphere, endosphere, and nodule compartments (Fig. S1, see online supplementary material). Plant compartments were the primary drivers of the root-associated microbiota composition (Fig. S1a; compartment, *R*^2^ = 0.576, *P* = 0.001; treatment, *R*^2^ = 0.008, *P* = 0.157). The community structure analysed by PCoA showed significant difference in the rhizosphere between the infested and noninfested plants (PERMANOVA; *P* < 0.05) (Fig. 1b). Infested plants exhibited lower microbial richness specifically in terms of rhizosphere bacteria (Fig. 1b; Kruskal–Wallis, *P* < 0.05). However, no significant difference in community structure and richness was observed between the infested and noninfested plants within the bulk soil, endosphere, and nodule compartments (Fig. 1a, c, and d). The microbial composition varied obviously across different ecological niches (Fig. 2a; Table S1, see online supplementary material). The dominant bacterial order in the bulk soil and rhizosphere compartments were Rhizobiales, Burkholderiales, Bacillales, Micrococcales, and Sphingomonadales, accounting for 83.37% and 69.27% of total relative abundance, respectively. The dominant bacterial order in the endosphere compartments were Rhizobiales, Burkholderiales, and Sphingomonadales, accounting for 64.54% of the total. The predominant order in the nodule compartment were Rhizobiales, accounting for 98.23% of the total. Moreover, leafminer infestation was found to increase the relative abundance of Rhizobiales in the rhizosphere and nodule compartments (Fig. 2a; Table S1, see online supplementary material). We further investigated the differences of the rhizosphere microbiota between the infested and noninfested plants at ASV level and examined the enrichment of ASVs based on their taxonomy using Manhattan plots. Comparing the microbiotas of infested and noninfested plants, we found the enriched ASVs in the infested plants encompassed a diverse group of bacterial phyla, including Acidobacteriota, Actinobacteriota, Bdellovibrionota, Chloroflexi, Desulfobacterota, Firmicutes, Planctomycetota, Proteobacteria, and Verrucomicrobiota (Fig. 2b). Notably, within the differential ASVs, we identified eight ASVs in the rhizosphere compartment were affiliated with Rhizobiales (Fig. 2c). Among these ASVs, one ASV belonging to *Mesorhizobium* and one ASV belonging to *Bradyrhizobium* in the rhizosphere compartment were enriched (Fig. 2c). *Mesorhizobium* and *Bradyrhizobium* have been reported to nodulate in cowpea [39]. These results indicated rhizobia were enriched in cowpea rhizosphere in response to leafminer infestation. To further assay the results, we also analysed the enrichment of rhizobia at genus level using metagenomic data. A total of 183 rhizobial genera were annotated in the rhizosphere compartment through metagenome sequencing, with *Bradyrhizobium*, *Rhizobium*, and *Mesorhizobium* being the predominant genera. Comparing the rhizobia microbiota in the infested plants to that in the noninfested plants, the enriched species were mostly from the genus *Bradyrhizobium*, whereas species from other rhizobial genera were mostly depleted in the infested plants (Fig. S2, see online supplementary material). To confirm the quantitative changes of *Bradyrhizobium* in rhizosphere, we analysed the absolute abundance of *Bradyrhizobium* in the infested plants and noninfested plants. Compared with control, the infested treatment exhibited a higher absolute abundance (Fig. S3, see online supplementary material, *t*-test, *P* < 0.001).

Overall, our findings indicate that leafminer infestation significantly influences the rhizosphere microbiota, primarily characterized by the enrichment of bacterial taxa belonging to the Rhizobiales clades.

### Leafminers destabilize rhizosphere microbiota co-occurrence networks

**Figure 3:**
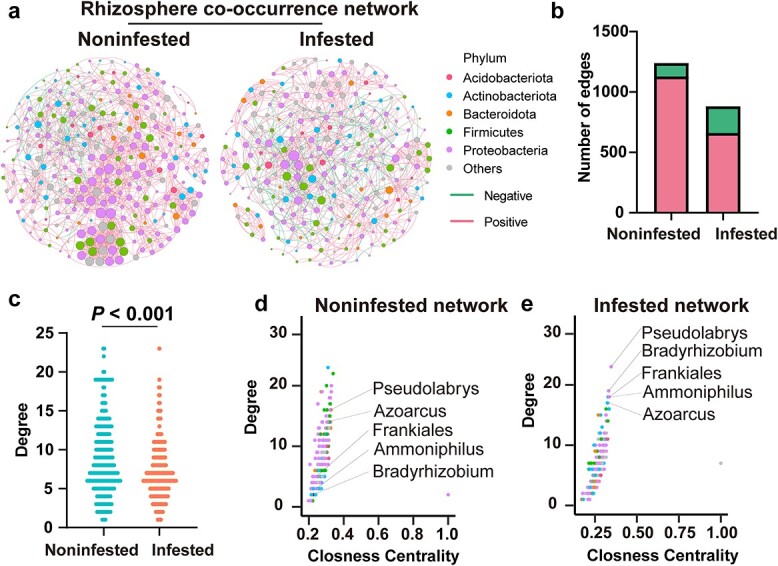
Co-occurrence networks of rhizosphere microbiotas in infested and noninfested plants. **a** Co-occurrence networks of rhizosphere noninfested or infested with leafminers. Each node represents a genus and is colored according to the phylum. The size of each node depends on its degree of connection. The red edges represent positive interactions, while the green edges represent negative interactions. The edges (**b**) and degree (**c**) of rhizosphere networks showing the lower network complexity in infested plants relative to noninfested plants. The significant difference of degree is assessed by Kruskal–Wallis test. Comparison of node-level topological features in noninfested network (**d**) and infested network (**e**) based on the degree and closeness centrality.

To further understand the impact of leafminer infestation on rhizosphere microbiota, we conducted the co-occurrence network analysis based on significant correlations (Spearman’s correlation test, *P* < 0.05) between the paired genera. This analysis allowed us to assess the topological properties of the networks and identify potential associations and key hubs within the rhizosphere microbiota responding to leafminer infestation. Leafminer infestation led to destabilization of the rhizosphere microbial networks (Fig. 3a). In particular, in the rhizosphere microbial networks, the average clustering coefficient decreased from 0.466 to 0.439, and the average path distance increased from 3.725 to 3.855 (Fig. 3a). The total number of edges in the microbial networks decreased from 1239 to 880, but the rate of negative edges increased from 8.96% to 25% (Fig. 3b). Moreover, the mean degree centrality decreased in the infested rhizosphere microbiota network relative to the noninfested rhizosphere microbiota network (Fig. 3c; Kruskal–Wallis test). To identify the most important microbes within the networks, we focused on ‘hubs’, which are highly connected microorganisms in the scale-free correlation networks [40]. We calculated the topological features, that is, degree and closeness centrality, for individual nodes. Interestingly, five taxa involved in N metabolism were selected as hubs in the rhizosphere networks with leafminer infestation (Fig. 3d and e). Notably, *Bradyrhizobium* [39], *Frankiales* [41], and *Azoarcus* [42] have been reported to participate in N fixation. Thus, these findings suggest that leafminer infestation destabilized the microbial networks of the rhizosphere microbiota, and several taxa associated with N metabolism emerged as hubs in the rhizosphere networks. Notably, *Bradyrhizobium*, which is capable of nodulating in cowpea, was enriched and selected as the one of the hub microbes by the plant rhizosphere.

### Leafminers affect rhizosphere microbiome function

Multiple microbiome characteristics from 16S rRNA sequencing analyses indicated a shift in rhizosphere microbiota on leafminer exposure (Figs. 1, 2, and 3). To further investigate the functional changes in the rhizosphere microbiome induced by leafminer infestation, metagenomic sequencing of rhizosphere microbiome from the infested and noninfested plants was performed. The rhizosphere samples from infested plants and noninfested plants yielded 163.8 Gbp of raw reads totally. After quality filtering and removing plant-derived sequences, we obtained 147.7 Gbp of microbial reads. Prediction of protein-coding genes from the assembly resulted in 18 821 241 putative genes, which were then clustered at 90% identity, generating 6 927 339 nonredundant genes.

**Figure 4:**
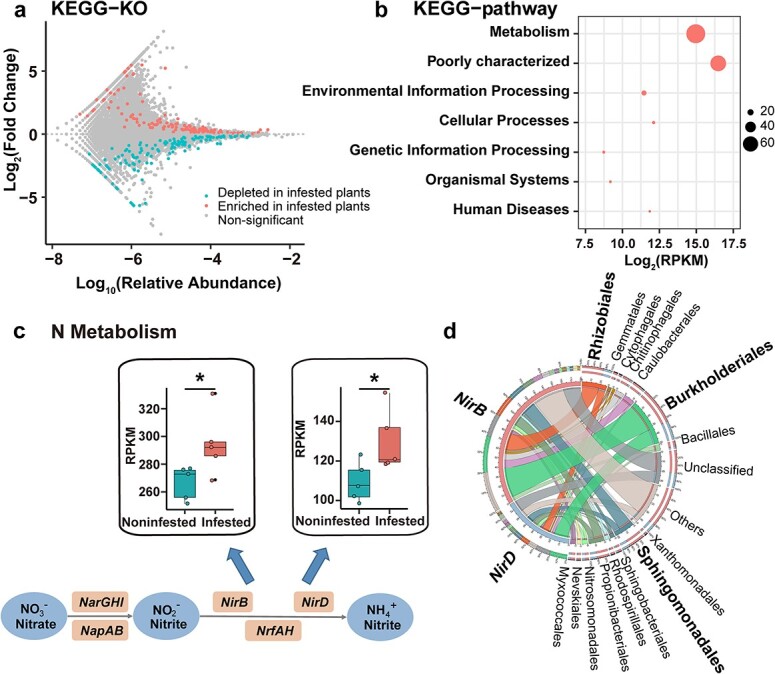
KEGG functional characterization of the rhizosphere microbiome in infested plants and noninfested plants. **a** Enriched and depleted KOs in infested plants. **b** The KEGG pathway with enriched KOs in the infested plants. The x-axis represents log_2_ transformed relative abundance of KOs. The circle sizes signify the number of enriched KOs in infested plants. **c** Differential KOs involved in N metabolism of rhizosphere microbiome in infested and noninfested plants. The diagram shows microbial pathways involved in the nitrogen metabolism. **d** Circos plot shows the distribution of *NirB* and *NirD* in rhizosphere microbiome. The width of the line represents the percentage of the distribution.

To estimate the effect of leafminer infestation on microbiome functional properties, we annotated the nonredundant genes using the KEGG database, resulting in 12 628 KOs. Differential enrichment analysis between the infested and noninfested plants identified 123 enriched KOs and 137 depleted KOs (Fig. 4a; Wilcoxon rank-sum test, *P* < 0.05). The majority of the enriched KOs were associated with metabolism pathways (Fig. 4b). In particular, the enriched KOs were involved in pathways of carbohydrate metabolism (15 KOs), amino acid metabolism (14 KOs), global and overview maps (9 KOs), lipid metabolism (8 KOs), metabolism of terpenoids and polyketides (7 KOs), energy metabolism (6 KOs), xenobiotics biodegradation and metabolism (6 KOs), metabolism of cofactors and vitamins (5 KOs), metabolism of other amino acids (3 KOs), biosynthesis of other secondary metabolites (1 KO), glycan biosynthesis and metabolism (1 KO), and nucleotide metabolism (1 KO) (Table S2, see online supplementary material). Notably, two KOs, namely *NirB* and *NirD* (K00362 and K00363), from N metabolism pathways (ko00910) were enriched in  rhizosphere microbiome of infested plants (Table S2, see online supplementary material). *NirB* and *NirD* encode assimilatory nitrite reductase responsible for the reduction from nitrite to ammonium [43], and their relative expression was higher in the rhizosphere microbiome of the infested plants than in that of the noninfested plants (Fig. 4c). To investigate the relationship between community members and gene functions, we analysed the functional contribution of community members to *NirB* and *NirD* using a Circos plot (Fig. 4d). We observed that Burkholderiales, Rhizobiales, and Sphingomonadales were the major contributors to both *NirB* and *NirD*, accounting for 29.6%–45.2% and 30.6%–48.52% of the total relative abundance, respectively (Fig. 4d). These findings suggest that the catalytic activity in ammonification process by Burkholderiales, Rhizobiales, and Sphingomonadales in the rhizosphere might play a potential role in the plant’s response to the aboveground leafminer infestation.

### *Bradyrhizobium* positively enhances plant performance and reduces leafminer fitness

**Figure 5:**
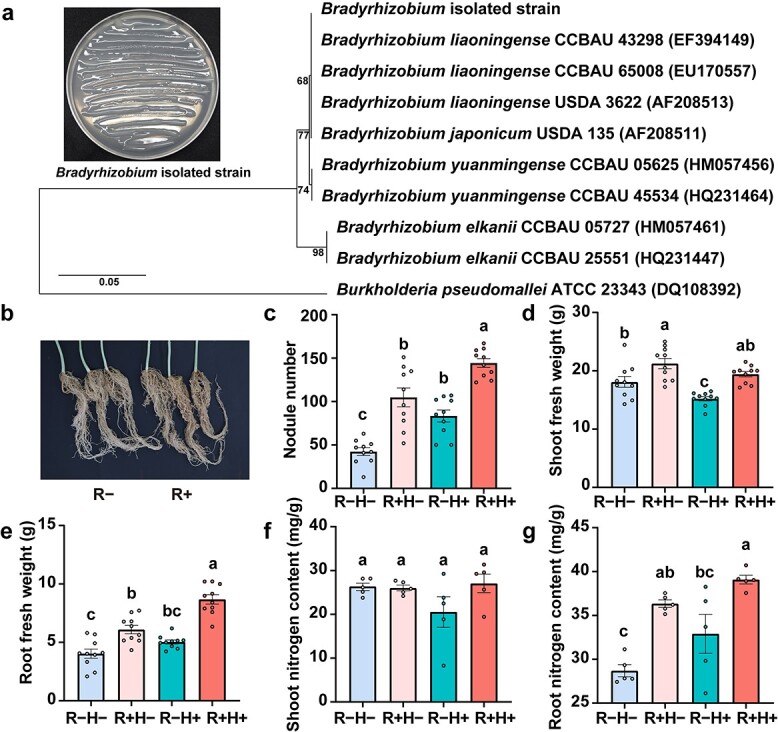
Growth performance of inoculated cowpea with leafminer infestation. **a** Neighbor-joining tree of *Bradyrhizobium* isolated strain. **b** Pictures showing cowpea roots inoculated without/with *Bradyrhizobium*. Cowpea performance in nodule number (**c**), shoot fresh weight (**d**), root fresh weight (**e**), shoot nitrogen content (**f**), and root nitrogen content (**g**) with different treatment. R−: without rhizobia; R+: with rhizobia; H−: without herbivory; H+: with herbivory. The data bars represent the means, while the error bars represent the standard error. Different letters denote significantly different treatments (*P* < 0.05, ANOVA, Tukey HSD).

**Figure 6:**
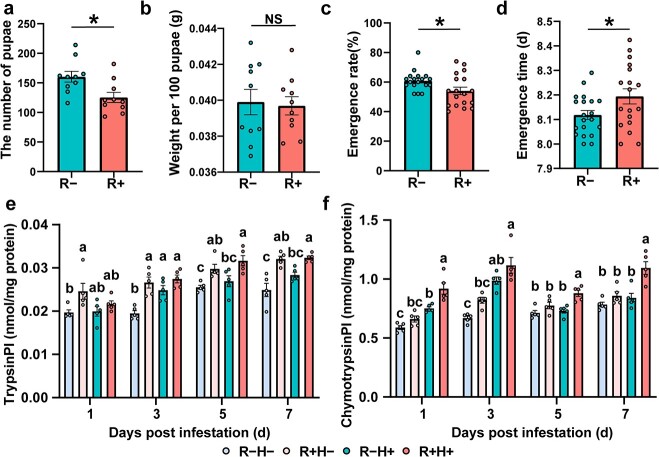
Plant defense of inoculated cowpea with leafminer infestation. The number of pupae (**a**), weight 100 pupae (**b**), emergence rate (**c**), and emergence time (**d**) of leafminer feeding on R− and R+ cowpea plants. Asterisks indicate significant differences between members of a treatment and control pair (*P* < 0.05, *t*-test). Trypsin protein inhibitor level (**e**) and chymotrypsin protein inhibitor level (**f**) with different treatment. Different letters indicate significantly different groups (*P* < 0.05, ANOVA, Tukey HSD).

To explore the role of *Bradyrhizobium* in plant–insect interaction, a *Bradyrhizobium* strain was isolated from the rhizosphere (Fig. 5a) and was proved to form nodules with cowpea (Fig. 5b). We evaluated the nodule number, shoot fresh weight, root fresh weight of four treatments: R − H−, R − H+, R + H− and R + H+; where ‘R−’ and ‘R+’ indicate the absence and presence of the rhizobial *Bradyrihobium*, and ‘H−’ and ‘H+’ indicate absence and presence of herbivory by leafminers, respectively (Fig. 5c, d, and e). Regardless of whether plants were challenged by herbivory, the nodule number, shoot fresh weight, root fresh weight were significantly higher in R+ (with rhizobia inoculation) plants compared to those from R− (without rhizobia inoculation) plants (Fig. 5c, d, and e; ANOVA, Tukey’s HSD, *P* < 0.05), indicating that the isolated strain could promote cowpea nodulation and growth. Interestingly, we found cowpea formed more nodules in R+ plants than that in R− plants with the challenge of leafminers, indicating that cowpea probably benefited from more nodules against leafminers (Fig. 5c). In the absence of rhizobia inoculation, the shoot fresh weight was significantly lower in H+ plants (with herbivory) plants compared to those from H− (without herbivory) plants (Fig. 5d), and no significant difference was observed in the root fresh weight (Fig. 5e). With the rhizobia inoculation, no significant difference was observed in the shoot fresh weight in H+ plants relative to H− plants (Fig. 5d), but the root fresh weight was significantly higher in H+ plants (Fig. 5e). These results suggested that *Bradyrhizobium* inoculation may have enhanced root performance to alleviate aboveground leafminer stress. Because multiple aspects of root performance were positively affected by *Bradyrhizobium*, we then tested whether this translated into greater nitrogen content in the shoots and roots with *Bradyrhizobium* inoculation. The results showed that *Bradyrhizobium* inoculation was associated with increased root nitrogen content (Fig. 5g; ANOVA, Tukey’s HSD, *P* < 0.05), but non-significant difference in the shoot nitrogen content (Fig. 5f; ANOVA, Tukey’s HSD, *P* < 0.05). Thus, a plausible assumption is that nitrogen contributed by *Bradyrhizobium* can allocate to the production of N-based defense chemical compounds in cowpea against leafminers. Firstly, to verify whether *Bradyrhizobium* inoculation can affect leafminer fitness, the number of pupae, weight per 100 pupae, emergence rate, and emergence time of leafminers feeding on R− and R+ plants were measured. The results showed that leafminers feeding on R+ plants exhibited a decrease in the number of pupae (Fig. 6a,  *t*-test, *P* < 0.05), a reduction in emergence rate (Fig. 6c,  *t*-test, *P* < 0.05), and an increase in emergence time (Fig. 6d,  *t*-test, *P* < 0.05). No significant difference was observed in the pupae weight of leafminers feeding on R− and R+ plants (Fig. 6d). These results suggested that leafminers feeding on the R+ plants would lead to a decrease in leafminer fitness.

Protease inhibitors are generally considered to be an important part of natural plant defense and are one of the N-based defense compounds. Over-expression of proteinase inhibitors results in increased resistance to *L. trifolii* in tomato [44]. In addition, it has been proved that protease inhibitors exhibit higher induction levels in plants supplied with high nitrogen compared to those supplied with low nitrogen [45]. Thus, we infer that nitrogen contributed by *Bradyrhizobium* allocate to the production of protease inhibitors against leafminers. To verify this hypothesis, we measured the content of two well-studied protease inhibitors in cowpea: trypsin protein inhibitor and chymotrypsin protein inhibitor [46]. In the absence of herbivory (H−), R+ plants had higher trypsin protease inhibitor levels relative to R− plants (Fig. 6e; ANOVA, Tukey’s HSD, *P* < 0.05), but no significant difference was observed in chymotrypsin protease inhibitor levels (Fig. 6f; ANOVA, Tukey’s HSD, *P* < 0.05). In the presence of herbivory (H+), R+ plants had higher trypsin protease inhibitor levels on 5 dpi (days post infestation) and 7 dpi, and higher chymotrypsin protease inhibitor levels on 1 dpi, 5 dpi, and 7 dpi (Fig. 6e and f), relative to R− plants. Together, our results demonstrated that *Bradyrhizobium* inoculation not only enhanced cowpea performance under leafminer stress but also increased protease inhibitor levels to decrease leafminer fitness.

## Discussion

Plant-associated microbiomes play a critical role in plant health and stress resistance in natural environments [3]. Evidence shows that plants use the ‘cry for help’ strategy combat multiple biotic and abiotic stresses [10], particularly in the rhizosphere compartments [4]. Recent advances in research on plant–microbe interactions have unraveled that plants can manipulate their root-associated microbiome to combat abiotic stresses and pathogens [7, 8, 12, 13, 47]; however, the role of the root-associated microbiome in plant–insect interactions remains relatively unexplored. In the present study, we investigated the changes in the root-associated microbiome of cowpea plants infested with aboveground leafminers (*L. trifolii*) and uncovered a shift in the rhizosphere microbiome that appears to aid plants in coping with insect herbivory. Our results suggest that changes in the cowpea rhizosphere microbiome in response to aboveground insect herbivory may feed back to reduce fitness of insect herbivores. These results help fill an important knowledge gap on the role of ‘cry for help’ strategies by plants in response to herbivore attack.

We observed significant differences in the community structure and taxa composition of the bulk soil, rhizosphere, endosphere, and nodule compartments (Fig. S1 and Table S1, see online supplementary material). A notable variation in β diversity and α diversity was observed in the rhizosphere when comparing infested plants with noninfested plants (Fig. 1b). This strong reaction in the rhizosphere compartment against leafminers can be attributed to the presence of several soil-borne microbes colonizing the plant rhizosphere to aid in plant defense against insects [24]. Aboveground herbivory reduced the photosynthetic rate of plant leaves, leading to nutrient imbalances, particularly that between C and N. This imbalance likely imposed a selective pressure on the cowpea rhizosphere microbiome, resulting in the reshaping of the microbial community in the rhizosphere of the infested plants (Figs. 1b and 2b). Within the differential ASVs in rhizosphere, several enriched ASVs were affiliated with Rhizobiales (Fig. 2c), indicating plants may call for more N from the rhizosphere microbiome in response to leafminer infestation. Results from co-occurrence network and KEGG pathway analyses of the rhizosphere microbiota also support this view (Figs. 3e and 4c).

Emerging studies on plant microbiomes have revealed that the root-associated microbiomes form complex, structured, and interconnected microbial networks and that the presence of microbial hubs may mediate the plant and microbiome responses to changing environments [40, 48]. Environmental stress affects not only the microbial communities but also the microbial interactions within a network structure [49]. Consistent with the results of previous researches, we found that leafminer infestation destabilized the rhizosphere networks and increased the proportion of negative correlations among microbes. This shift toward negative correlations (Fig. 3a and b) suggests that the competition among microbes may be intensified in response to leafminer infestation [50]. Host plants may benefit from the competition among microbes, resulting in enhanced resistance to environmental stress [51]. Several microbes involved in N metabolism were identified as hubs in the rhizosphere networks following leafminer infestation (Fig. 3b). Hubs, which are highly connected microorganisms in a network, exert a strong influence on microbial communities [40]. Environmental factors can directly affect hub microbes, which then transmit these environmental effects to the entire microbial community via microbe–microbe interactions [40]. Therefore, our study suggests that plants actively select microbes involved in N metabolism to be hubs in the rhizosphere when they are exposed to insect herbivores.

Our analysis revealed that several enriched and hub microbes in the cowpea rhizosphere, particularly those involved in N metabolism, may exhibit plant growth-promoting activities in response to leafminer infestation (Figs. 2c and 3). In particular, some species within the *Frankiales*, *Bradyrhizobium*, and *Azoarcus* taxa are well-known N-fixing bacteria [39, 41, 42]. The enrichment of functional categories related to metabolism further supported the involvement of nutrient acquisition-based interactions in the plant rhizosphere following leafminer infestation (Fig. 4). The rhizosphere-enriched microbial functions associated with N metabolism, specifically in the ammonification process, were prominently contributed by species within the Burkholderiales, Rhizobiales, and Sphingomonadales taxa (Fig. 4c and d). Previous studies have identified these taxa as plant growth-promoting rhizobacteria that colonize the plant rhizosphere [52]. This finding suggests that the rhizosphere of infested plants may exhibit enhanced efficiency in N transformation processes, which could enhance the overall performance of plants against leafminer infestation. It has been reported that certain crops, such as maize, enrich specific microbes in the Oxalobacteraceae taxon in the rhizosphere to enhance N capture under N-deprived conditions [47]. Similarly, rice plants manipulate the root-associated microbiome to acquire N under natural conditions for optimal plant growth [6], indicating that the root-associated microbiome could alleviate nutritional stress on crops. Moreover, the additional N provided by the root-associated microbiome could be translocated to the shoot and utilized for the production of N-based defense compounds, including glucosinolates, cyanogenic glycosides, alkaloids, various peptides, proteins, and nonprotein amino acids, thereby contributing to herbivore control [53, 54]. Therefore, we hypothesize that when plants are exposed to insect herbivores, they enrich microbes that aid in N utilization and facilitate the translocation of N from the rhizosphere to the aboveground parts of the plant, thus alleviating herbivory-induced stress. While we hypothesize that the microbiome changes are due to the enrichment, it remains possible that certain bacteria are favored while others are disfavored, or that the multiplication of specific bacteria outcompetes others within the existing microbiome. The existing evidences have indicated root exudates act as signaling molecules, inhibitors, repellents, attractants and stimulants, exerting a multitude of effects on rhizosphere microbes [55]. These suggest that the microbiome changes are due to a variety of reasons and these changes could be mediated by root exudates. However, to the best of our knowledge, current research related to plant-associated microbiome focuses on the role of microbial enrichment in response to environmental stress, and no reports found on the disfavoring/out-competing of specific bacteria [6, 9, 12, 13, 47]. These potential mechanisms warrant further investigation to fully understand the dynamics underlying the observed changes in the microbiome.

Studies have indicated that certain soil-borne microbes can be manipulated to induce plant resistance to affect aboveground insects’ performance [23]. Of these, rhizobia inoculation plays a crucial role in protecting legumes against insect herbivores [22, 56, 57]. The cyanogenic clover strains inoculated with *Rhizobium leguminosarum* have been reported to affect the development of the chewing herbivore *Spodoptera littoralis* larvae [57]. Similarly, *Rhizobium* inoculation was found to enhance the production of cyanogenic defense compounds in lima bean, resulting in reduced herbivore chewing [22]. Inoculation with *Bradyrhizobium japonicum* decreases the population of aphids in soybean [56]. The nitrogen contributed by rhizobia is assigned to the production of N-based defense chemical compounds, and hence influence plant resistance to insect herbivores [22, 56, 58]. In conclusion, the plant–rhizobia interactions not only promote plant growth but also enhance plant defense against herbivores. Thus, we infer that the cowpea rhizosphere actively enrichs rhizobia to enhance plant performance and defense against leafminers. Our evidence suggested that inoculating cowpea plants with the *Bradyrhizobium* isolated strain positively enhanced cowpea performance to mitigate the effects of herbivory stress (Fig. 5c, d, and e) and increased root nitrogen content to affect protease inhibitor levels to decrease leafminer fitness (Figs. 5g and 6).

## Conclusions

Our study revealed that aboveground leafminers significantly reshaped the cowpea rhizosphere microbiome. The rhizosphere responded by enriching N metabolism-related microbes to promote plant growth and enhance plant defense against leafminers. Our study provides novel insights on plant–microbe–herbivore interactions as well as the evidence to support the ‘cry for help’ strategy in plant–insect interactions. The interaction among the rhizosphere microbiome, herbivores, and host plant traits may promote the development of novel strategies to manipulate the rhizosphere microbiome for improving crop productivity and sustainability.

## Materials and methods

### Soil preparation

The soil used in this experiment was collected from a field located in Sanya, China (109°09′26.965′′ N, 18°21′19.011′′ E). The field has been used for natural crop cultivation for over a decade. After collection, the soil was transported to the laboratory and air-dried at room temperature. It was then pooled together and sieved through a 3-mm sieve to remove any plant tissue and rocks. The soil comprised 82.1% sand, 17.6% silt, and 0.3% clay, with a pH of 5.6. Additionally, the soil analysis conducted by the South China Botanical Garden, Chinese Academy of Science, revealed the nutrient contents as follows: total N, 0.60 g/kg; total phosphorus, 31.4 g/kg; and total potassium, 0.396 mg/kg.

### Plant and insect materials

The cowpea (*V. unguiculata*) cultivar ‘Haiyate’ was purchased from Jiangxi Huanong Seed Industry Co. Ltd (Jiangxi, China). To prepare the seeds, they were subjected to surface sterilization, which involved immersing them in 75% ethanol for 30 s and 5% NaClO for 5 min. They were then soaked overnight in sterile water. The sowing of cowpea seeds was carried out in plastic trays filled with our prepared soil mixture. The trays were placed in a climate-controlled room under the following conditions: light intensity of 100 μmol m^2^ s^−1^, a photoperiod of 16 h:8 h (light:dark), temperature of 28°C, and relative humidity set at 70%. After 10 days, two plantlets were transplanted into separate pots (top diameter × height × bottom diameter = 15 × 14 × 10 cm) containing 2.3 kg of soil. The pots were placed in the growth chamber randomly and watered every 2 days.

A laboratory colony of the American serpentine leafminer, *L. trifolii*, was maintained on potted cowpea plants inside insect cages (length × width × height = 50 × 50 × 50 cm) at 25 ± 2°C, under a 16:8 h (light:dark) photoperiod and 65% relative humidity. Pupae were collected from an insect cage, placed in Petri dishes, and kept in another cage until adult emergence under the same laboratory conditions as our plants.

### Experimental design and sample collection

Our experimental treatments included cowpea plants infested (treated) and noninfested (control) with leafminers. Each treatment included 10 replicate cages with 2 plants per cage. The cages dimension were length × width × height = 25 × 25 × 50 cm. Plants were 3 weeks old at the start of the experiment and were watered every 2 days. Infestation involved the release of 10 mated female adult leafminers into each of the treated 10 cages, whereas no leafminers were released into the control cages noninfested. Before leafminer infestation, the soil was covered with plastic wrap to prevent pupae from falling into the soil. Infested and noninfested plants were randomly placed in the climate room. Oviposition and feeding punctures caused by gravid female adult flies resulted in leaf damage, which was visible after a few hours. Approximately 2 days after infestation, the eggs hatched into larvae that fed within the leaves, damaging the mesophyll tissue and creating visible mining tunnels. Our experiment ran for 7 days post-infestation, after which most mature larvae dropped from the foliage and pupated.

Bulk soil, rhizosphere, endosphere, and nodule samples were collected from the root systems of infested and noninfested plants following the protocol by Zhang [59] with some modifications. Bulk soil samples were collected from 1 cm below the surface without roots in the pots. To collect root samples, the loose soil was manually removed by shaking the entire roots vigorously, leaving approximately 1 mm of root-adhered soil particles. For the separation of roots and rhizosphere soil, two roots with soil particles adhering to them from each pot were placed in a sterile 50-mL tube containing 30 mL of 0.1 M phosphate-buffered saline (PBS) (per liter: 80 g of NaCl, 35.8 g of Na_2_HPO_4_·12H_2_O, 2.59 g of KH_2_PO_4_, and 2.0 g of KCl; pH 7.4). The mixture was then vibrated at the maximum speed for 15 s, and plant tissue and large sediments were filtered out using a sterile 50-mL tube with a 100-μm nylon mesh cell strainer. The turbid filtrate was subsequently centrifuged at 10000 x *g* for 5 min, and the resulting tight pellet at the bottom of the tube was collected as the rhizosphere sample. For endosphere and nodule sampling, the filtered roots were repeatedly washed with PBS until the buffer became clear after vortexing. The roots were then transferred to a new sterile 50-mL tube containing 30 mL of PBS and sonicated at 60 Hz for 3 min (30 s of sonication followed by a 30-s break for three cycles). Then nodules were cut off with sterile scissors. Bulk soil, rhizosphere, endosphere, and nodule samples were snap-frozen in liquid N and stored at −80°C until analysis. The samples collected from the infested cages were designated as the infested group, whereas those from the noninfested cages were designated as the noninfested group. Eight sample types, each comprising 10 biological replicates, were prepared for 16S rRNA gene amplicon sequencing, and two additional rhizosphere sample types, each consisting of five biological replicates, were prepared for shotgun metagenomic sequencing.

### 16S rRNA gene amplicon sequence processing and statistical analyses

To explore the variations in the root-associated microbiota between infested and noninfested plants, we collected bulk soil, rhizosphere soil, endosphere, and nodule samples. Bacterial DNA was extracted from bulk soil, rhizosphere, endosphere, and nodule samples. The 16S ribosomal RNA (rRNA) gene sequencing of the V5–V7 region was performed to generate microbial community profiles for the samples. The 16S rRNA V5–V7 region was amplified using the primer pairs 799F and 1193R [59]. The amplicons were sequenced on the Illumina MiSeq PE300 platform. The bacterial 16S rRNA gene sequences were demultiplexed, quality filtered with fastp (0.19.6) [60], and merged using FLASH (v1.2.11) [61]. Subsequently, the high-quality sequences, referred to as amplicon sequence variants (ASVs), were denoised using the Diverse Amplicon Denoising Algorithm 2 (DADA2) [38] plugin in the Qiime2 [62] (version 2020.2) pipeline with recommended parameters. Each ASV was then taxonomically classified based on comparisons with the SILVA 138 database. The Kruskal–Wallis test was used to assess statistical differences in alpha diversity of microbial communities between the infested and noninfested plants in different compartments. PCoA with Bray–Curtis distance was employed to evaluate the similarity of microbial communities across different samples, whereas permutational multivariate analysis of variance was used to assess beta diversity differences among the sample groups. The statistical significance of relative abundance differences at ASV level between plants was determined using the Wilcoxon rank-sum test.

A candidate species identified from our analysis was *Bradyrhizobium*. We therefore performed additional validation of *Bradyrhizobium* absolute abundance (Biomaker Technologies Co. Ltd, Beijing, China). The sequencing and bioinformatics analysis was conducted according to the previous methods [63].

### Metagenomic sequencing and statistical analysis

To further explore the influence of infestation in rhizosphere microbiome, we used metagenomic sequencing to provide complimentary microbiome community data to our 16S sequencing for a subset of our samples. DNA extracts from five infested rhizosphere samples and five noninfested rhizosphere samples were subjected to pair-end sequencing on the Illumina NovaSeq. The raw data was processed and trimmed using fastp [60]. To eliminate any potential contamination from the host plant, cleaned reads were aligned against the *V. unguiculata* genome (accession: ASM 411807v2) [64] using BWA [65]. The remaining metagenomic reads were assembled using MEGAHIT [66], and contigs shorter than 300 bp were discarded. Open reading frames were predicted from the assembled contigs using MetaGene [67] in the metagenomics mode, and nonredundant gene sets were generated with CD-HIT [68] at a similarity threshold of 95%. Taxonomic annotations were assigned to the non-redundant genes by aligning them against the NR database [69], whereas functional annotations were performed by aligning the genes against the KEGG database [70] using Diamond [71]. Differences in KO relative abundance were evaluated using the Wilcoxon rank-sum test.

### Co-occurrence network analyses

The microbial co-occurrence networks were established by calculating the genus-level relative abundance correlations to explore the internal community relationships among the samples [72]. If the *P*-value was <0.05 and Spearman’s correlation coefficient (*ρ*) was >0.70, the co-occurrence was considered robust: that is, two genera significantly co-occur. Gephi 0.9.2 was used to visualize the networks [73]. In the microbial network, nodes represent specific microbial genera, and edges represent biologically significant or biochemically relevant interactions between node pairs. Various topological characteristics of the networks, such as the numbers of positive and negative edges, average path length, average degree, modularity, and average clustering coefficient were calculated in Gephi. The role of each node depends on their topological features of closeness centrality and degree in the entire networks [40], which were also calculated in Gephi. The Kruskal–Wallis test was employed to determine the statistics difference in degree of rhizosphere network between the groups.

### Isolation and characterization of rhizobia

To isolate rhizobia from cowpea rhizosphere, 10 g rhizosphere soil was put into 90 mL sterile water and then shaken for 20 min. Soil suspension was streaked onto yeast mannitol agar (YMA) medium. The culture plate was incubated at 28°C for 10 days and monitored daily to isolate the desired colonies. The isolates were then purified through three rounds of subculturing, and 16S rRNA gene was amplified by the primer pairs 27F and 1492R. The obtained 16S rRNA gene sequence was submitted to the NCBI (https://www.ncbi.nlm.nih.gov/) with the GenBank accession number OQ942632. The phylogenetic tree of the identified bacteria was constructed using MEGA 11 software. The neighbor-joining method was employed for tree construction, and the reliability of the tree was evaluated through bootstrap analysis.

### Plant growth assays

To investigate the growth promotion effects of rhizobia under leafminer infestation, *Bradyrhizobium* inoculum was applied to soil pots. The isolated strain was cultured in yeast mannitol broth (YMB) medium and incubated at 28°C to OD600 = 1.0. Cowpea plants were grown as previously described and subjected to four different conditions: (i) 10 mL sterile water per pot without leafminers, representing the R − H− treatment; (ii) 10 mL inoculum per pot without leafminers, representing the R + H− treatment; (iii) 10 mL sterile water per pot with leafminer infestation, representing the R − H+ treatment; and (iv) 10 mL inoculum per pot with leafminer infestation, representing the R + H+ treatment. Each treatment had 10 replicates. For inoculation, 10 mL inoculum or sterile water was applied to 1-week-old plants prior to infestation. After 2 weeks of growth, ten *L. trifolii* mating pairs were introduced into an insect cage containing a single plant, allowing them to puncture and oviposit on the plant. Following 1 week of infestation, the mature larvae naturally dropped from the foliage, and the remaining larvae were carefully removed using tweezers. Subsequently, the plants were harvested, and the number of nodules, fresh root weight, fresh shoot weight, root nitrogen content and shoot nitrogen content were determined. One-way analysis of variance (ANOVA), followed by Tukey’s honestly significant difference (HSD) test, was used to compare the plant traits among the treatments.

### Herbivore growth assays

To assess the fitness of leafminer feeding on the R− and R+ plants, ten *L. trifolii* mating pairs were released to the insect cage to infest the 3-weeks-old plants and were removed after 24 h. The number of pupations was observed and recorded every night at 8 p.m. and per 100 pupae were weighed. Then every 50 pupae were placed in a clear glass dish, and the number of emergence was observed and recorded at 8 p.m. every day. Finally, the effect on insect fitness was quantified as the number of pupae, weight per 100 pupae, emergence rate and emergence time. Differences between treatment means were estimated by *t*-test.

### Trypsin protein inhibitor and chymotrypsin protein inhibitor analysis

To investigate the influence of rhizobia addition and leafminer infestation on the content of protease inhibitor in plant leaves, we employed four different treatments, including R − H−, R + H−, R − H+ and R + H+ treatment. Each treatment had five replicates. Samples were collected at 1 d, 3 d, 5 d, and 7 d after infestation and immediately frozen for subsequent assay. Intermediate leaves were chosen for sampling. The uppermost leaf was defined as young leaves, the leaves between young and old leaves were defined as intermediate leaves, and old leaves referred to the first two leaves of each plant. Each sample weighing 100 mg was mixed with 900 μl PBS solution, followed by centrifugation at 2000 x *g* for 15 minutes. The resulting supernatant was carefully collected for further analysis using the plant trypsin inhibitor ELISA kit and plant chymotrypsin inhibitor ELISA kit (Zoman Bio-tek, Jiangsu, China). The statistical difference of protease inhibitor content among the treatments was evaluated by ANOVA and HSD test.

## Acknowledgements

This work was financially supported by Hainan Province Science and Technology Special Fund (ZDYF2021XDNY302), Hainan Provincial Natural Science Foundation of China (323RC521), Sanya Science and Technology Innovation Project (2022KJCX19), Hainan Special PhD Scientific Research Foundation of Sanya Yazhou Bay Science and Technology City (HSPHDSRF-2022-04-010). We thank Prof. Hongye Li (Zhejiang University) and Ming Zhang (Zhejiang University) for their insightful discussions and Dr Joshua A. Thia (The University of Melbourne) for his effort on reviewing and professional English editing.

## Author contributions

Y.G., Z.-R.Z., and Yi.Z. designed the experiments; Y.G., Q.C., Y.H., W.H., and J.G. performed the experiments; Y.G. wrote the manuscript. Z.-R.Z., Yi.Z., Yu.Z., and Q.Y. revised the manuscript.

## Data availability

The sequencing raw data have been uploaded to the National Genomics Data Center (https://ngdc.cncb.ac.cn/gsa) [74, 75] with accession number GSA: CRA013220.

## Conflict of interest statement

All authors declare that no conflict of interest exists.

## Supplementary data

Supplementary data is available at *Horticulture Research* online.

## Supplementary Material

Web_Material_uhae121
