# Supplementary material for: Plants attacked above-ground by leaf-mining flies change below-ground microbiota to enhance plant defense
Source: Hortic Res. 2024 Apr 26;11(6):uhae121. doi: 10.1093/hr/uhae121 (PMC11197306; doi:10.1093/hr/uhae121)
Supplement: Web_Material_uhae121 [file web_material_uhae121.docx]

**Title:**

**Plants attacked above-ground by leaf-mining flies change below-ground microbiota to enhance plant defense**

**Running head:**

**Below-ground microbiota enhance plant defense**

**Authors:**

Yang Gao^1,2^, Qiong Yang^3^, Qiulin Chen^1,2^, Yunchuan He^1,2^, Wei He^1,2^, Jiamei Geng^1,2^, Yunzeng Zhang^4^, Ying Zhou^1*^, Zeng‑Rong Zhu^1,2^

^1^Hainan Institute, Zhejiang University, Sanya, 572025, China

^2^State Key Laboratory of Rice Biology, Institute of Insect Sciences, Zhejiang University, Hangzhou, 310058, China

^3^School of BioSciences, Bio21 Institute, The University of Melbourne, Parkville, VIC 3010, Australia

^4^Joint International Research Laboratory of Agriculture and Agri‐product Safety of the Ministry of Education, Yangzhou University, Yangzhou, 225009, China

***Corresponding authors:**

Ying Zhou (yzhyzb@ zju.edu.cn)

**Supplementary Figure 1: Community structure and microbial diversity in different compartments.** **a**, Unconstrained PCoA (for principal coordinates PCo1 and PCo2) with Bray‑Curtis metrics was conducted to characterize the beta diversity and analyzed in PERMANOVA to test for differences. **b**, Shannon index of the bacterial community in different compartments. Different letters indicate significantly different groups (*P* < 0.05, ANOVA, Tukey HSD).


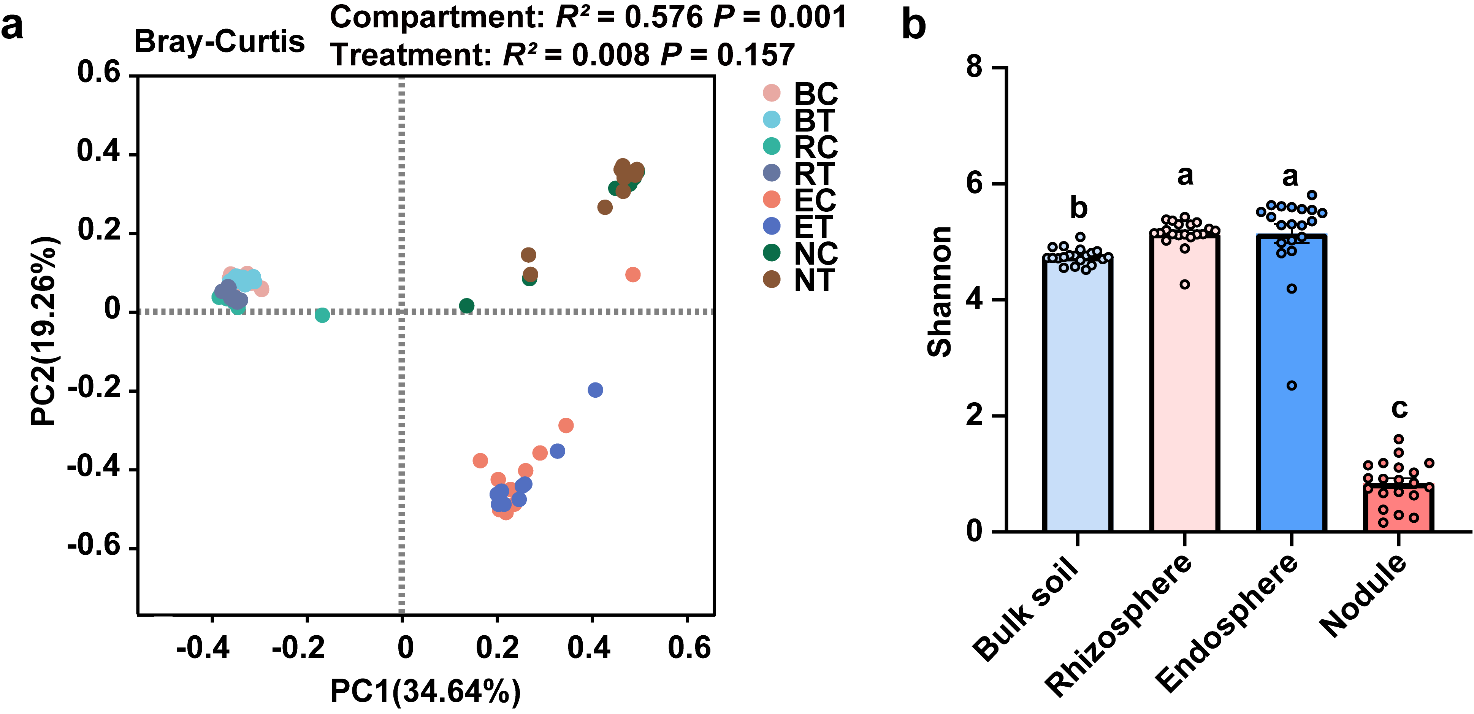


**Supplementary Figure 2: Manhattan plot showing Rhizobiales species enriched or depleted in the infested plants in rhizosphere compartments according to metagenome sequence.** Each circle or triangle represents a single species. Species enriched or depleted in the infected plants are represented by filled or empty triangles (*P* < 0.05, Wilcoxon rank-sum test).

**
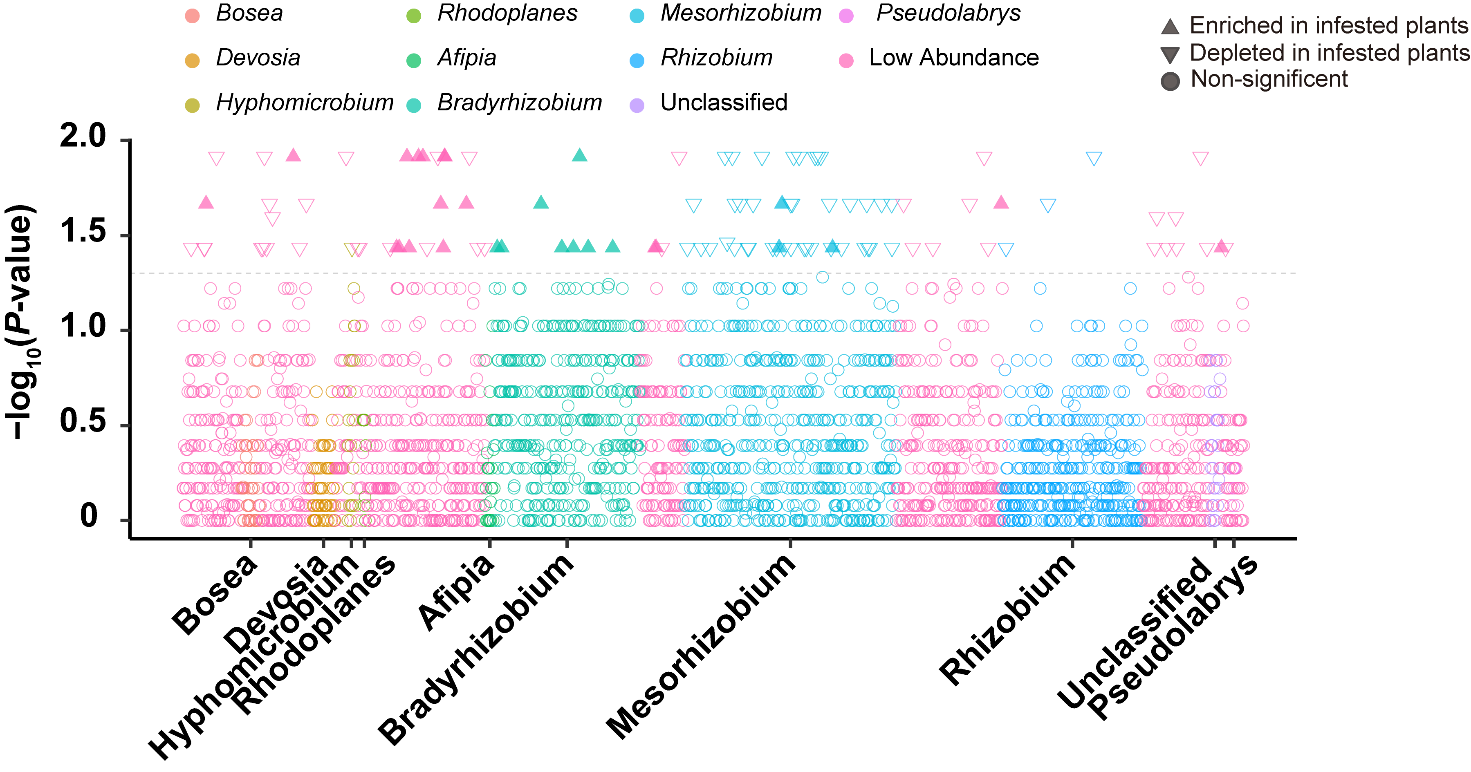
**

**Supplementary Figure 3: Absolute abundance of *Bradyrhizobium* in infested plants and noninfested plants in rhizosphere compartments.** R: rhizosphere; C: noninfested plants; T: infested plants. The significance level of different treatment was assessed by *t* test.

**
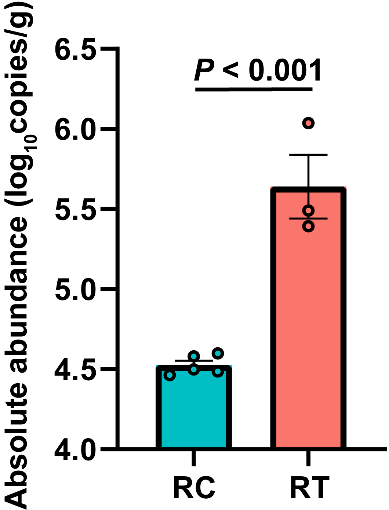
**

| **Supplementary Table 1: Order table of root-associated microbiota.** | | | | | | | | |
| --- | --- | --- | --- | --- | --- | --- | --- | --- |
| **Order** | **BC** | **BT** | **RC** | **RT** | **EC** | **ET** | **NC** | **NT** |
| Rhizobiales | 8.5327% | 8.4296% | 40.6404% | 33.3922% | 97.2200% | 99.2381% | 11.7397% | 13.3039% |
| Burkholderiales | 33.9701% | 26.5293% | 20.0577% | 28.6713% | 1.2980% | 0.3975% | 26.7427% | 22.9506% |
| Bacillales | 27.7230% | 36.6618% | 0.6110% | 0.5042% | 0.0037% | 0.0012% | 22.3138% | 26.1281% |
| Micrococcales | 9.2173% | 6.3379% | 1.7286% | 1.6943% | 0.0098% | 0.0135% | 4.3406% | 3.8265% |
| Sphingomonadales | 5.2264% | 4.1038% | 3.2683% | 3.0401% | 0.0417% | 0.0515% | 3.8731% | 3.3113% |
| Others | 15.3306% | 17.9377% | 33.6940% | 32.6978% | 1.4268% | 0.2981% | 30.9901% | 30.4797% |

| **Supplementary Table 2: The number of enriched‑KOs in infested samples in rhizosphere at different level of KEGG pathway.** | | | | |
| --- | --- | --- | --- | --- |
| **Pathway ID** | **Level 1** | **Level 2** | **Level 3** | **The number of enriched‑KOs** |
| ko00380 | Metabolism | Amino acid metabolism | Tryptophan metabolism | 3 |
| ko00330 | Metabolism | Amino acid metabolism | Arginine and proline metabolism | 3 |
| ko00360 | Metabolism | Amino acid metabolism | Phenylalanine metabolism | 2 |
| ko00310 | Metabolism | Amino acid metabolism | Lysine degradation | 2 |
| ko00260 | Metabolism | Amino acid metabolism | Glycine serine and threonine metabolism | 2 |
| ko00280 | Metabolism | Amino acid metabolism | Valine leucine and isoleucine degradation | 1 |
| ko00250 | Metabolism | Amino acid metabolism | Alanine aspartate and glutamate metabolism | 1 |
| ko00940 | Metabolism | Biosynthesis of other secondary metabolites | Phenylpropanoid biosynthesis | 1 |
| ko00650 | Metabolism | Carbohydrate metabolism | Butanoate metabolism | 3 |
| ko00640 | Metabolism | Carbohydrate metabolism | Propanoate metabolism | 2 |
| ko00500 | Metabolism | Carbohydrate metabolism | Starch and sucrose metabolism | 1 |
| ko00620 | Metabolism | Carbohydrate metabolism | Pyruvate metabolism | 1 |
| ko00030 | Metabolism | Carbohydrate metabolism | Pentose phosphate pathway | 1 |
| ko00040 | Metabolism | Carbohydrate metabolism | Pentose and glucuronate interconversions | 1 |
| ko00562 | Metabolism | Carbohydrate metabolism | Inositol phosphate metabolism | 1 |
| ko00630 | Metabolism | Carbohydrate metabolism | Glyoxylate and dicarboxylate metabolism | 1 |
| ko00052 | Metabolism | Carbohydrate metabolism | Galactose metabolism | 1 |
| ko00051 | Metabolism | Carbohydrate metabolism | Fructose and mannose metabolism | 1 |
| ko00053 | Metabolism | Carbohydrate metabolism | Ascorbate and aldarate metabolism | 1 |
| ko00520 | Metabolism | Carbohydrate metabolism | Amino sugar and nucleotide sugar metabolism | 1 |
| ko00190 | Metabolism | Energy metabolism | Oxidative phosphorylation | 2 |
| ko00910 | Metabolism | Energy metabolism | Nitrogen metabolism | 2 |
| ko00920 | Metabolism | Energy metabolism | Sulfur metabolism | 1 |
| ko00680 | Metabolism | Energy metabolism | Methane metabolism | 1 |
| ko01212 | Metabolism | Global and overview maps | Fatty acid metabolism | 3 |
| ko01200 | Metabolism | Global and overview maps | Carbon metabolism | 3 |
| ko01220 | Metabolism | Global and overview maps | Degradation of aromatic compounds | 2 |
| ko01230 | Metabolism | Global and overview maps | Biosynthesis of amino acids | 1 |
| ko00513 | Metabolism | Glycan biosynthesis and metabolism | Various types of N-glycan biosynthesis | 1 |
| ko00071 | Metabolism | Lipid metabolism | Fatty acid degradation | 2 |
| ko00061 | Metabolism | Lipid metabolism | Fatty acid biosynthesis | 2 |
| ko00600 | Metabolism | Lipid metabolism | Sphingolipid metabolism | 1 |
| ko00564 | Metabolism | Lipid metabolism | Glycerophospholipid metabolism | 1 |
| ko01040 | Metabolism | Lipid metabolism | Biosynthesis of unsaturated fatty acids | 1 |
| ko00592 | Metabolism | Lipid metabolism | alpha-Linolenic acid metabolism | 1 |
| ko00860 | Metabolism | Metabolism of cofactors and vitamins | Porphyrin and chlorophyll metabolism | 2 |
| ko00750 | Metabolism | Metabolism of cofactors and vitamins | Vitamin B6 metabolism | 1 |
| ko00130 | Metabolism | Metabolism of cofactors and vitamins | Ubiquinone and other terpenoid-quinone biosynthesis | 1 |
| ko00670 | Metabolism | Metabolism of cofactors and vitamins | One carbon pool by folate | 1 |
| ko00430 | Metabolism | Metabolism of other amino acids | Taurine and hypotaurine metabolism | 1 |
| ko00473 | Metabolism | Metabolism of other amino acids | D-Alanine metabolism | 1 |
| ko00410 | Metabolism | Metabolism of other amino acids | beta-Alanine metabolism | 1 |
| ko00909 | Metabolism | Metabolism of terpenoids and polyketides | Sesquiterpenoid and triterpenoid biosynthesis | 2 |
| ko00903 | Metabolism | Metabolism of terpenoids and polyketides | Limonene and pinene degradation | 2 |
| ko00253 | Metabolism | Metabolism of terpenoids and polyketides | Tetracycline biosynthesis | 1 |
| ko01057 | Metabolism | Metabolism of terpenoids and polyketides | Biosynthesis of type II polyketide products | 1 |
| ko01053 | Metabolism | Metabolism of terpenoids and polyketides | Biosynthesis of siderophore group nonribosomal peptides | 1 |
| ko00230 | Metabolism | Nucleotide metabolism | Purine metabolism | 1 |
| ko00627 | Metabolism | Xenobiotics biodegradation and metabolism | Aminobenzoate degradation | 3 |
| ko00622 | Metabolism | Xenobiotics biodegradation and metabolism | Xylene degradation | 1 |
| ko00643 | Metabolism | Xenobiotics biodegradation and metabolism | Styrene degradation | 1 |
| ko00361 | Metabolism | Xenobiotics biodegradation and metabolism | Chlorocyclohexane and chlorobenzene degradation | 1 |
| - | Poorly characterized | Poorly characterized | Poorly characterized | 62 |
| ko02010 | Environmental Information Processing | Membrane transport | ABC transporters | 3 |
| ko02060 | Environmental Information Processing | Membrane transport | Phosphotransferase system(PTS) | 3 |
| ko02020 | Environmental Information Processing | Signal transduction | Two-component system | 7 |
| ko04024 | Environmental Information Processing | Signal transduction | cAMP signaling pathway | 1 |
| ko04070 | Environmental Information Processing | Signal transduction | Phosphatidylinositol signaling system | 1 |
| ko02024 | Cellular Processes | Cellular community - prokaryotes | Quorum sensing | 4 |
| ko02025 | Cellular Processes | Cellular community - prokaryotes | Biofilm formation - Pseudomonas aeruginosa | 1 |
| ko04111 | Cellular Processes | Cell growth and death | Cell cycle - yeast | 1 |
| ko04146 | Cellular Processes | Transport and catabolism | Peroxisome | 1 |
| ko03018 | Genetic Information Processing | Folding sorting and degradation | RNA degradation | 1 |
| ko03420 | Genetic Information Processing | Replication and repair | Nucleotide excision repair | 1 |
| ko03022 | Genetic Information Processing | Transcription | Basal transcription factors | 1 |
| ko03040 | Genetic Information Processing | Transcription | Spliceosome | 1 |
| ko03008 | Genetic Information Processing | Translation | Ribosome biogenesis in eukaryotes | 2 |
| ko04212 | Organismal Systems | Aging | Longevity regulating pathway - worm | 1 |
| ko03320 | Organismal Systems | Endocrine system | PPAR signaling pathway | 1 |
| ko04922 | Organismal Systems | Endocrine system | Glucagon signaling pathway | 1 |
| ko04725 | Organismal Systems | Nervous system | Cholinergic synapse | 1 |
| ko04727 | Organismal Systems | Nervous system | GABAergic synapse | 1 |
| ko01501 | Human Diseases | Drug resistance: Antimicrobial | beta-Lactam resistance | 1 |
| ko01503 | Human Diseases | Drug resistance: Antimicrobial | Cationic antimicrobial peptide (CAMP) resistance | 1 |
| ko04940 | Human Diseases | Endocrine and metabolic diseases | Type I diabetes mellitus | 1 |
| ko05150 | Human Diseases | Infectious diseases: Bacterial | Staphylococcus aureus infection | 1 |

| **Supplementary Table 3: Functional contribution at order level to NirB and NirD.** | | | | | | | | | | | | |
| --- | --- | --- | --- | --- | --- | --- | --- | --- | --- | --- | --- | --- |
| **Gene** | **Order** | **RC1** | **RC2** | **RC3** | **RC4** | **RC5** | **RT1** | **RT2** | **RT3** | **RT4** | **RT5** | **Total** |
| NirB | Burkholderiales | 12.54% | 17.71% | 12.95% | 21.83% | 18.67% | 17.22% | 24.64% | 13.50% | 13.90% | 29.25% | 18.47% |
| NirB | Rhizobiales | 7.01% | 12.06% | 10.76% | 11.70% | 13.19% | 7.87% | 12.14% | 10.45% | 12.72% | 8.16% | 10.53% |
| NirB | Sphingomonadales | 5.81% | 10.63% | 8.12% | 7.93% | 10.79% | 11.42% | 6.56% | 5.65% | 8.23% | 7.69% | 8.27% |
| NirB | Propionibacteriales | 2.81% | 5.19% | 3.96% | 7.29% | 6.09% | 6.09% | 4.31% | 4.14% | 8.98% | 10.17% | 6.02% |
| NirB | Caulobacterales | 5.33% | 5.59% | 5.10% | 7.61% | 5.53% | 5.59% | 4.66% | 4.69% | 5.70% | 4.15% | 5.37% |
| NirB | unclassified_p__Bacteroidota | 1.58% | 2.08% | 3.99% | 2.87% | 1.75% | 3.43% | 2.75% | 2.28% | 2.83% | 1.31% | 2.48% |
| NirB | unclassified_c__Alphaproteobacteria | 4.92% | 3.80% | 5.55% | 5.24% | 3.51% | 5.28% | 2.25% | 5.12% | 5.09% | 1.96% | 4.23% |
| NirB | Chitinophagales | 2.04% | 1.82% | 2.55% | 2.49% | 2.65% | 3.18% | 4.43% | 4.73% | 8.48% | 3.94% | 3.68% |
| NirB | Bacillales | 1.53% | 2.70% | 2.62% | 2.10% | 0.99% | 2.96% | 2.80% | 2.83% | 2.93% | 7.14% | 2.97% |
| NirB | Nitrosomonadales | 2.90% | 2.63% | 2.28% | 1.73% | 1.99% | 2.52% | 1.61% | 1.32% | 1.11% | 1.28% | 1.92% |
| NirB | Myxococcales | 5.13% | 1.94% | 3.77% | 1.76% | 1.14% | 0.88% | 0.88% | 3.22% | 0.72% | 0.65% | 1.96% |
| NirB | unclassified_p__Acidobacteria | 3.32% | 2.74% | 2.94% | 0.30% | 1.62% | 3.02% | 1.06% | 3.75% | 1.02% | 1.04% | 2.05% |
| NirB | Sphingobacteriales | 3.12% | 0.93% | 1.18% | 1.99% | 2.62% | 1.30% | 1.38% | 1.89% | 2.77% | 1.43% | 1.84% |
| NirB | Gemmatales | 4.25% | 2.30% | 1.76% | 1.46% | 2.17% | 1.18% | 1.16% | 2.18% | 1.31% | 1.66% | 1.92% |
| NirB | Nevskiales | 1.71% | 1.83% | 3.82% | 0.98% | 1.77% | 2.27% | 1.35% | 3.54% | 0.54% | 0.49% | 1.79% |
| NirB | Rhodospirillales | 1.80% | 1.48% | 3.13% | 1.55% | 2.15% | 1.32% | 2.95% | 0.68% | 1.02% | 2.31% | 1.85% |
| NirB | unclassified_p__Proteobacteria | 0.78% | 2.02% | 1.23% | 1.46% | 2.60% | 2.08% | 2.50% | 0.62% | 1.23% | 1.58% | 1.61% |
| NirB | unclassified_c__Acidobacteriia | 2.82% | 0.46% | 2.92% | 0.92% | 0.60% | 1.18% | 1.22% | 1.54% | 0.64% | 0.23% | 1.23% |
| NirB | Xanthomonadales | 0.56% | 1.42% | 0.77% | 2.70% | 0.22% | 1.67% | 1.04% | 0.46% | 0.80% | 0.39% | 1.00% |
| NirB | Cytophagales | 3.06% | 0.35% | 1.15% | 0.42% | 0.66% | 0.91% | 1.05% | 2.10% | 0.84% | 0.47% | 1.09% |
| NirB | Others | 26.99% | 20.31% | 19.45% | 15.66% | 19.27% | 18.63% | 19.28% | 25.31% | 19.13% | 14.68% | 19.73% |
| NirD | Burkholderiales | 17.70% | 21.45% | 14.28% | 19.67% | 23.26% | 16.85% | 35.28% | 10.58% | 16.48% | 31.95% | 21.16% |
| NirD | Rhizobiales | 8.22% | 9.68% | 8.88% | 10.64% | 13.15% | 10.62% | 8.50% | 11.65% | 11.20% | 8.63% | 10.11% |
| NirD | Sphingomonadales | 7.49% | 14.72% | 11.21% | 11.36% | 9.66% | 9.91% | 8.71% | 8.38% | 7.46% | 7.94% | 9.61% |
| NirD | Propionibacteriales | 3.65% | 6.13% | 5.01% | 12.00% | 5.54% | 9.23% | 3.72% | 9.62% | 11.03% | 9.45% | 7.74% |
| NirD | Caulobacterales | 3.21% | 3.37% | 1.72% | 4.96% | 4.56% | 5.88% | 2.11% | 3.08% | 2.35% | 2.99% | 3.46% |
| NirD | unclassified_p__Bacteroidota | 4.72% | 7.69% | 17.71% | 7.05% | 7.15% | 13.90% | 10.11% | 14.01% | 10.72% | 8.16% | 10.19% |
| NirD | unclassified_c__Alphaproteobacteria | 4.09% | 3.73% | 2.42% | 1.37% | 0.80% | 4.30% | 2.33% | 2.63% | 2.10% | 0.51% | 2.36% |
| NirD | Chitinophagales | 3.59% | 1.61% | 1.22% | 1.63% | 2.41% | 1.92% | 4.04% | 4.67% | 8.26% | 4.00% | 3.37% |
| NirD | Bacillales | 1.29% | 0.54% | 1.66% | 0.28% | 0.11% | 1.77% | 0.60% | 0.76% | 1.48% | 12.47% | 2.45% |
| NirD | Nitrosomonadales | 4.25% | 6.34% | 2.59% | 4.87% | 7.12% | 2.90% | 2.97% | 5.29% | 2.26% | 1.97% | 3.94% |
| NirD | Myxococcales | 5.95% | 3.11% | 7.79% | 1.83% | 5.04% | 1.33% | 2.53% | 6.79% | 4.87% | 0.54% | 3.76% |
| NirD | unclassified_p__Acidobacteria | 1.93% | 1.75% | 3.15% | 1.30% | 0.99% | 1.93% | 2.04% | 4.57% | 1.44% | 1.06% | 1.98% |
| NirD | Sphingobacteriales | 2.14% | 0.39% | 1.43% | 2.84% | 0.60% | 1.72% | 1.67% | 1.04% | 3.61% | 1.12% | 1.65% |
| NirD | Gemmatales | 0.55% | 1.51% | 2.08% | 0.81% | 0.80% | 0.87% | 1.51% | 1.19% | 0.83% | 0.15% | 0.99% |
| NirD | Nevskiales | 0.00% | 1.94% | 2.04% | 0.61% | 1.24% | 1.17% | 0.12% | 0.58% | 0.00% | 0.23% | 0.77% |
| NirD | Rhodospirillales | 0.18% | 0.33% | 1.34% | 0.36% | 0.53% | 0.00% | 0.32% | 0.00% | 0.56% | 0.43% | 0.40% |
| NirD | unclassified_p__Proteobacteria | 0.41% | 0.33% | 0.12% | 0.62% | 4.35% | 0.00% | 0.80% | 0.00% | 0.41% | 0.14% | 0.69% |
| NirD | unclassified_c__Acidobacteriia | 2.76% | 0.93% | 1.16% | 0.40% | 1.55% | 2.30% | 0.37% | 2.05% | 2.80% | 1.04% | 1.52% |
| NirD | Xanthomonadales | 0.85% | 1.93% | 2.43% | 4.40% | 0.57% | 2.78% | 3.17% | 0.45% | 2.23% | 0.44% | 1.92% |
| NirD | Cytophagales | 3.71% | 1.83% | 0.89% | 1.57% | 0.49% | 0.45% | 0.69% | 2.21% | 1.25% | 1.01% | 1.34% |
| NirD | Others | 23.30% | 10.70% | 10.85% | 11.40% | 10.07% | 10.16% | 8.40% | 10.48% | 8.66% | 5.77% | 10.58% |
